# Effect of Carbon Nanofibers on Physical, Adhesion and Rheological Properties of Liquid Epoxidized Natural Rubber Modified Asphalt

**DOI:** 10.3390/ma15113870

**Published:** 2022-05-29

**Authors:** Ramez A. Al-Mansob, Herda Yati Katman, Abdulnaser M. Al-Sabaeei, Muhammad Zamzami, Amin Al-Fakih, Willy Kuay Wei, Taha M. Jassam, Jamal Alsharef, Salihah B. Surol, Nurul H. Yusof, Suhana Koting

**Affiliations:** 1 Department of Civil Engineering, UCSI University, Cheras 56000, Malaysia or ramez@iium.edu.my (R.A.A.-M.); danazamzam1@gmail.com (M.Z.); willykuaywei@gmail.com (W.K.W.); taha@ucsiuniversity.edu.my (T.M.J.); salihah@ucsiuniversity.edu.my (S.B.S.); 2 Department of Civil Engineering, International Islamic University Malaysia, Jalan Gombak 50728, Selangor, Malaysia; 3 Institute of Energy Infrastructure, Universiti Tenaga Nasional, Putrajaya Campus, Jalan IKRAM-UNITEN, Kajang 43000, Selangor, Malaysia; 4 Department of Civil and Environmental Engineering, Universiti Teknologi PETRONAS, Seri Iskandar 32610, Malaysia; 5 Centre for Research in Data Science, Universiti Teknologi PETRONAS, Seri Iskandar 32610, Malaysia; 6 Interdisciplinary Research Center for Construction and Building Materials, King Fahd University of Petroleum and Minerals, Dhahran 31261, Saudi Arabia; aminali.fakih@kfupm.edu.sa; 7 Department of Civil Engineering, Wadi Alshatti University, 68 Brak Alshati, Wadi Alshatti, Libya; j.alshref@wau.edu.ly; 8 Civil Engineering Department, Faculty of Engineering, Tripoli University, Tripoli, Libya; 9 LGM, Engineering and Technology Division, Malaysian Rubber Board, Petaling Jaya 47000, Malaysia; hayati@lgm.gov.my; 10 Center for Transportation Research, Department of Civil Engineering, Faculty of Engineering, University of Malaya, Kuala Lumpur 50603, Malaysia; suhana_koting@um.edu.my

**Keywords:** carbon nanofibers, liquid epoxidized natural rubber, polymer, rheology, asphalt

## Abstract

This study aimed to evaluate the effects of carbon nanofibers (CNFs) on the performance of liquid epoxidized natural rubber (LENR)-modified asphalt. The physical, adhesion and rheological properties were determined by several tests such as penetration, elastic recovery, ring and ball softening point, Brookfield rotational viscometer, AFM and dynamic shear rheometer. LENR was used at concentrations of 3, 6, and 9%, while CNFs were used at contents of 0.3, 0.4, and 0.5% by weight of asphalt. Conventional test results showed that the increases in LENR and LENR/CNFs composite contents in binder leads to an increase in the hardness and consistency and a reduction in the temperature susceptibility of base asphalt. Adhesion results revealed that the addition of CNFs significantly increases the adhesion and bonding properties of base and rubberized binders. Rheological properties analysis exhibited that LENR improved the viscoelastic properties and permanent deformation resistance of asphalt at different temperatures and frequencies. On the other hand, it was found that the addition of CNFs significantly improves the stiffness, elasticity, and hardness of LENR-modified binders. The 6% LENR and 0.4% CNFs were found to be the optimum to enhance the physical, adhesion, and rheological properties of asphalt in this study. Thus, it can be stated that the addition of CNFs is promising to improve the performance of rubberized binders for high temperature applications.

## 1. Introduction

For hundreds of years, asphalt as an engineering material has been used in the construction industry for its unique viscoelastic properties. Bituminous materials are broadly used for the various construction applications such as preservative, sealant, adhesive, waterproofing agent, and pavement binder [1]. Millions of metric tons of asphalt materials are produced worldwide annually and there is a continued increase in global demand [2]. However, asphalt has limited strength and elasticity and can be lacking in performance and can lead to exposing various distresses such as rutting, fatigue, moisture damage, and low-temperature cracks when subject to extreme traffic loading and/or environmental changes [3]. Therefore, pavement industries and researchers are interested in the modification of asphalt using various polymers to improve its performance for wide range of pavement situations.

Polymer-modified asphalt (PMA) is developed by integrating one or more types of polymers into base asphalt based on the targeted construction applications. New and advantageous physical, chemical, rheological, and microstructural properties can be obtained by integrating asphalt with a polymer [4,5]. It is well known that the behaviour of pavement materials is highly affected by the traffic and climate and most of the road pavement structures are subjected to axial load transferred by highly complex stresses [6]. Therefore, the main objective of polymer-modified asphalt is to increase the pavement life service. Styrene butadiene styrene (SBS) is classified as a thermoplastic rubber and it is one of the most ordinary polymers used for modified asphalt despite having an economic and significant technical limitation [7]. According to the previous research, it was reported that the performance of SBS-modified asphalt mainly depends on the source of asphalt, compatibility of the asphalt with polymer, and the polymer concentration. It was stated that at the high content of SBS polymer with high aromatic asphalt binder, the results are a highly elastic network in the asphalt matrix that increases the viscosity, complex modulus, and elastic response of the PMA, particularly at high temperatures [5].

According to Zhang et al. [8], styrene butadiene rubber (SBR) is also considered one of the most common polymers for asphalt modification. It was found that SBR can stiffen the binder and enhance the aggregate bonding toward decreasing the stripping. SBR also brings the advantage of aging stability by decreasing the change of material properties due to oxidation. At the same time, SBR can improve the modified asphalt’s elastic recovery and ductility that results in adequate flexibility and resistance of asphalt materials to cracks in cold weather regions. Ethylene-vinyl acetate (EVA) is another polymer used for asphalt modification known for its improvement of the compatibility of polymers with base asphalt binders [9]. However, another study claimed that the addition of EVA into asphalt binder could affect the temperature sensitivity of the modified binder [10].

Crumb rubber (CR) is another form of the common polymers that are used for asphalt modification due to its chemical interaction in bitumen matrix. CR-modified asphalt binders and mixtures are used at different places around the world to address quality problems such as permanent deformation distresses [3]. It was also claimed that CR has the ability to improve the fatigue and aging resistance and durability of asphalt binders and mixtures due to the improvement of binder stiffness, ductility, and film thickness of rubberized binders compared to base asphalt [11,12,13]. In addition, the use of rubber waste in asphalt pavement contributes to mitigate the noise of asphalt pavement and the environmental pollution due to the disposal of rubber waste in a nonproper manner [12,13]. On the other hand, the applications of conventional CR in asphalt binder and mixtures modification have their own disadvantages such as the need for long time mixing at high temperatures which leads to increasing the required energy and produces high emission. However, in the end, rubber particles cannot be dissolved totally in asphalt and that results in an incompatibility issue, poor storage stability, and poor workability, which has a direct effect on the behaviour and performance of asphalt binders and mixtures [2,14,15]. Overall, the use of rubber in a vulcanized state, such as crumb rubber, may cause a problem with the difficulty of dispersing the polymer and producing a homogenous binder, and it is required to be mixed at high temperature and certain long duration mixing [1]. Additionally, it was found that the multiphase structure of asphalt rubber significantly changed during the aging process due to the degradation of rubber particles. It was also reported that there is a significant effect of aging conditions on the swelling rubber that is due to the stiffening influence of swelling rubber at high temperature and low frequencies and its softening influence at low temperature and high frequencies are both weakened after aging [16]. Therefore, researchers and industries have begun looking for another alternative to address the disadvantages of conventional rubber and one of the sustainable alternatives that has been investigated is natural rubber.

Natural rubber (NR) is one of the most available polymers worldwide and it is used as an asphalt modifier, alongside its usage to produce a wide variety of daily commodities such as swim caps, gloves, balloons, and mattresses [17]. According to the reliable literature, it was found that the optimum NR that can improve the performance of asphalt binders is around 5% by weight of asphalt [18,19]. Epoxidized natural rubber (ENR), as a modified NR polymer, is chemically altered natural rubber with additional proxy formic acid that has been investigated as an alternative to base natural rubber. ENR exhibits high strength with the ability to bear strain crystallization and improve glass transition temperature which made this material a good binder. As an asphalt modifier, ENR showed an improvement in the viscosity, storage, and complex modulus of asphalt binders [20,21]. It was reported that the optimum ENR percentage content is 6% to be used in asphalt modification, with a proven improvement in storage ability and temperature susceptibility of binders. Besides, ENR provides the binder with an elastic network that leads to increment stiffness, viscosity, and elastic behaviour of the binder. These lead to enhancement of the rutting and fatigue characteristic of the ENR-modified asphalt binders [20,21].

Liquid epoxidized natural rubber (LENR) is a new form of ENR that is produced by breakdown of the high molecular weight of ENR into short length chain. Due to the limited solubility and processability of ENR, different methods can be used to produce the LENR such as chemical degradation with the presence of potassium peroxidisulphate, mechanical milling, and photo-oxidation with the help of ultraviolet radiation [22]. Contrary to ENR, LENR has several benefits in producing multiple products because it takes less energy and is simpler to process. In addition, LENR can be easily modified due to the lower molecular weight [23]. In conjunction with, for example, its sticky shape, high elasticity, and strength, LENR shows special physical and mechanical properties. Those are the main factors in enhancing the composites or polymer blends’ durability and flexibility. On the other hand, some polymers including rubber are shown to be thermodynamically incompatible to be used as the asphalt modifier due to differences between the asphalt and polymer properties [1]. Thus, many researchers are working on improving the limitation of polymer-modified asphalt.

The use of nanomaterials is one of the most common method to improve the compatibility of asphalt binders with the polymers [3,24]. Overall, it can be said that researchers have focused on nanotechnology as an additive in asphalt binders for its significant improvement of the characteristics and performance of asphalt pavement [25]. It was also reported that using nanomaterials as a modifier for asphalt binders leads to enhancing the storage stability, aging resistance, thawing attack, durability, and cost efficiency in terms of maintenance [26]. Nowadays, nanomaterials have gained popularity and the potential to improve the performance and durability of construction materials. Significant improvement has been shown in the high-temperature, intermediate temperature, lower temperature, and moisture damage resistance performance of asphalt binders and mixtures as a results of modifying with nanomaterials [27]. The huge surface area, desired shape and size, adhesion, polarity, and chemical interaction properties of most of the nanomaterials make them one of the best alternatives to improve the wide range of properties of based, modified, and composite asphalt binders [27]. Nanoalumina (Al_2_O_3_), nanosilica (SiO_2_) nanoclay, carbon nanotubes (CNTs), and carbon nanofibers (CNFs) are the most popular nanomaterials being used in asphalt modification.

You et al. [28] employed the nanoclay, which is one of the popular nanomaterials that is being used in asphalt modification, as a modifier to evaluate the effects on asphalt binders and mixtures performance. It was found that nanoclay is able to reduce the strain failure and increase the shear complex modulus of the binder. Moreover, it showed an improvement for moisture damage resistance of the asphalt mixture. Similarly, Muniandy et al. [29] conducted a study to investigate the effect of organic montmorillonite nanoclay (OMMT) on the physical and rheological properties of asphalt binder. It was reported that the physical and rheological properties of base asphalt were significantly enhanced due to the increase in the complex shear modulus and decrease in phase angle that led to higher rutting resistance compared to the control binder. Recent research has been carried out by Al-Mansob et al. [21] to study the effects of nano-alumina (Al_2_O_3_) as an additive on the physical, rheological, and compatibility of ENR-modified asphalt binders. Results showed a remarkable improvement in the compatibility of base asphalt with ENR, that led to adequate storage stability and dispersion of rubber particles in the asphalt matrix. Meanwhile, it was reported that physical and rheological properties of base and ENR-modified binders also improved as a result of the increasing of viscosity, stiffness, adhesion, and elastic properties with the presence of nanomaterial.

Carbon nanotubes (CNTs) are another widely used nanomaterial in asphalt modification because of their lightweight, strength properties, large surface area, and desired particle size. Ul Haq et al. [30] conducted a research to study the effects of CNTs on the thermal and rheological performance of asphalt. It was concluded that the addition of CNTs into asphalt decreases the thermal susceptibility. As well, increasing CNTs content resulted in increasing the complex shear modulus and decreasing the phase angle due to the improvement of elastic behaviour, stiffness of binders, and permanent deformation resistance with the presence of CNTs. These findings of the effects of CNTs were supported by another study conducted by Gong et al. [31]. Recently, carbon nanofibers (CNFs) are shown an interest among the asphalt pavement researchers for their improvement of rutting and fatigue performances of asphalt materials [32]. S. G. Jahromi [33] conducted an experimental study to evaluate the effects of CNTs on the performance of asphalt mixtures. It was found that CNTs exhibited consistency in results (stability, voids volume, resilient modulus, dynamic creep, and fatigue life). CNTs had suitable effects on properties of asphalt mixtures by decreasing the flow value and increasing its stability and voids volume while decreasing the permanent strain (dynamic creep) as the CNTs content increased. The fatigue life of mixtures increased up to 48.2%. As a result, the stability, permanent deformation resistance, and fatigue cracking resistance of the asphalt mixture have been improved due to the addition of CNTs. It was also concluded that CNTs are a promising nanomaterial for improving the mechanical performance of asphalt binders and mixtures at various loading and environmental conditions.

However, most of the aforementioned nanomaterials have been investigated to improve the mechanical properties of conventional crumb rubber-modified asphalt binders, but no study has been reported to evaluate the interaction effects of CNTs and LENR on the physical, rheological, and adhesion properties of asphalt binders. It can also be stated that LENR polymer is a new polymer and has never been studied before as an asphalt modifier. Therefore, in this study, three different binders have been evaluated: base asphalt as a control, LENR-modified asphalt (LENRMA), and carbon nanofiber (CNF)-modified LENRMA. The main objective was to evaluate the effects of LENR and CNTs separately and in composite on the mechanical properties of asphalt binder. In this study, LENR was used as a biopolymer to improve the elastic properties of asphalt binder, while CNFs were used as the catalyst to enhance the compatibility of LENR in asphalt binder matrix to come up with better temperature sensitivity resistance, consistency, adhesion, and viscoelastic properties of the base and LENR-modified binders.

## 2. Materials and Methods

### 2.1. Materials

**Figure 1:**
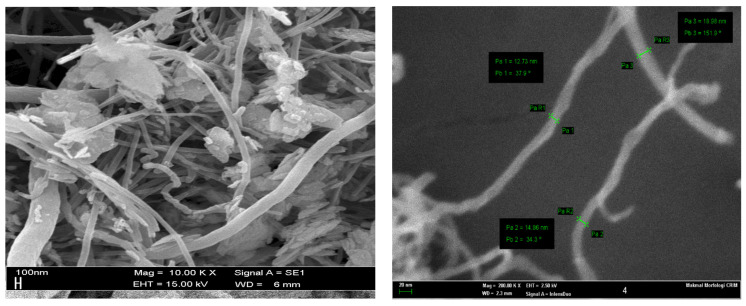
Transmission electron microscopy (TEM) images of CNFs.

The materials used in this study were asphalt, liquid epoxidized natural rubber (LENR) and carbon nanofibers (CNFs). The 60/70 penetration grade asphalt binder was supplied by ACP-DMT SDN BHD (MTD Group), Malaysia. LENR was provided by the Malaysian Rubber Board. Table 1 shows the properties of the base asphalt, LENR, and CNFs. Figure 1 shows the transmission electron microscopy of CNFs.

### 2.2. Preparation of Modified Binders

The LENR content percentages in the base asphalt that were used were 0, 3, 6, and 9% by weight of asphalt. To prepare the liquid epoxidized natural rubber-modified asphalts (LENRMAs), the asphalt was first heated to an easily flowable consistency. Upon reaching 140 °C, the LENR was incorporated slowly into the base asphalt and mixed by using a high shear mixer for 30 min with 4000 rpm. Each of the samples was labelled accordingly based on the polymer content. For example, a sample of modified asphalt with nine percent of LENR content was coded as LENRMA9. Additionally, the three different contents of CNFs used were 0.3, 0.4, and 0.5% by weight of base asphalt binder and mixed with the best content of LENR to come up with a composite LENR/CNFs asphalt binders. Composite binders were coded as PMN0.3, PMN0.4, and PMN0.5. CNFs and CNFs/LENR composite binders were prepared at 160 °C with 4000 rpm for two hours. These mixing parameters are the most commonly used in the literature for modifying asphalt binders with nanomaterials [3]. Content of LENR was chosen based on the previous studies of using solid epoxidized natural rubber (ENR) as a modifier in asphalt binders [19,20,21,34,35]. Similarly, the CNF contents in this study were chosen according to previous studies [32,33]. After preparing the modified asphalt binders, they were evaluated based on conventional binder tests (penetration, softening point, elastic recovery and viscosity), the rheological test was performed using a dynamic shear rheometer (DSR), and atomic force microscopy (AFM) was used to study the adhesion properties of the binders. At least three replicates were tested for each binder and each test. Table 2 illustrates the identifications (IDs) of the binders in this study.

### 2.3. Conventional Physical Properties

Conventional tests such as penetration, ring and ball softening point, rotational viscosity, and elastic recovery tests of base and modified binders were conducted according to the American Society for Testing and Materials Standards (ASTM).

The penetration grade that indicates the hardness and consistency of asphalt binder at intermediate service temperatures can be verified by a penetration test. The experiment was conducted according to ASTM D5 standard [36]. A penetrometer was used to measure the penetration value of melted samples at 25 ± 0.5 °C, where a standard needle penetrated the sample. Softer binder produces a higher value of penetration.

Due to the viscoelastic properties of asphalt, it gets softer and less viscous as its temperature increases. Therefore, an arbitrary and closed specific method known as ring and ball softening point was conducted according to ASTM D36 standard [37]. The ring and ball softening point test can be used to determine which temperature is causing the binder to have phase change or become less viscous.

The fluid viscousness and the flow characteristic of the asphalt binder have been evaluated using rotational viscosity test. The fluid viscosity is defined as internal resistance to the fluid flow. The viscosity of binders was tested using the Brookfield Model DV-III in accordance with ASTM D4402 standard [38]. A rotating spindle type viscometer was used at 80 to 200 °C in this study.

### 2.4. Temperature Susceptibility

The sensitivity to temperature of the binder in terms of penetration index (*PI*) can be calculated based on the penetration test and softening point test results. The increasing of *PI* is an indicator of the improvement of temperature susceptibility resistance of binders while a lower *PI* value indicates the poor temperature susceptibility resistance of binders. By using the simplified formula referred to in the Shell Bitumen Handbook [1], *PI* can be identified.

(1) $$PI=\frac{1952-500\times \log\left(pen_{25}\right)-20\times SP}{50\times \log\left(pen_{25}\right)-SP-120}$$

where *SP* is the value of the softening point in degrees (°C) and *pen*_25_ is the penetration value at 25 °C. A *PI* value of the modified asphalt that ranges from approximately −3 to −7 indicates high temperature susceptibility and approximately positive 3 to 7 indicate low temperature susceptibility.

The penetration viscosity number (PVN) was obtained by testing the viscosity of binders at 135 °C and the penetration value at 25 °C. Equation (2) was used to calculate the PVN for base and modified binders in this study.

(2) $$\mathrm{PVN}=\frac{\log L-\log X}{\log L-\log M}\times -1.5$$

where *X* is the logarithm of viscosity in millipascal, *L* = 4.25800 − 0.79670 log $pen_{25}$ and *M* = 3.46289 − 0.61094 log $pen_{25}$. Furthermore, it was reported that the lower the value of PI and PVN, the more the asphalt is susceptible to temperature.

### 2.5. Elastic Recovery

To measure the elasticity of the modified bitumen, an elastic recovery test was conducted. The experiment was carried out in accordance with the ASTM D6084 standard [39] at 25 °C. The elasticity test may be perceived as an indirect measure of the tensile properties of the material. The calculation for recovery percentage was found using the following formula:

(3) $$\mathrm{Recovery},\;\%=\frac{E-X}{E}\times 100$$

where *E* is the original elongation of sample in cm and *X* is the elongation of the specimen with severed ends just touching in cm.

### 2.6. Atomic Force Microscopy

Atomic force microscopy (AFM) is a specific type of scanning probe microscopy (SPM), where a sharp probe (tip radius = 10 nm) located near the end of a cantilever beam scans across a sample surface using piezoelectric scanners. In this study, NT-MDT SPM AFM was used to evaluate the adhesion of binders. All samples were kept at 25 °C for two hours prior to testing. Measuring the interaction forces between the tip and the surface of the sample was achieved with the usual calibration process to transform experimental cantilever deflection curves as a function of vertical scanner displacement Dx into force–distance curves. The slope of the retraction deflection curve was used in the contact region, and then the cantilever deflection was converted into force using Hooke law:

F = k∆x(4)

where F is the atomic force, k = 22 is the stiffness constant of the AFM cantilever, and ∆x is the distance between the probe–sample contact point and the disengage point.

### 2.7. Dynamic Shear Rheometer

Asphalt is a viscoelastic material, and its stiffness is a function of the stress and strain. To identify the rheological properties of binders, the Rheometer HAAKE RheoStress 600 model of the dynamic shear rheometer (DSR) from Thermo Electron Corporation was used in this study in accordance with ASTM D7175 [40]. The rheological properties of both base and modified binders were tested within the leaner viscoelastic region at a temperature range between 20 and 80 °C and frequencies of 0.1 to 10 Hz. A parallel geometry with 1 mm gap and 25 mm diameter was used for a temperature range of 40 to 80 °C, while 2 mm gap and 8 mm in diameter for a temperature range of 20 to 40 °C. The linear viscoelastic region (LVER) limit was specified when the complex modulus (G*) decreased to 95% of its zero strain. The shear was determined based on the torque applied or calculated, and the asphalt binder’s dimension. The complex modulus (G*) and phase angle (δ) of the tested binders were determined by exposing the samples to dynamic shear stress. The G* and δ were used to determine the rutting or permanent deformation parameters.

### 2.8. Statistical Analysis

User-defined design (UUD) is one of the common statistical and mathematical approaches of response surface methodology (RSM) that is used to evaluate the relationship between the independent and dependent variables. In this research, the design and statistical analysis was conducted using Design-Expert software, version 10.0.8. The independent variables that were considered in this research were the LENR at four levels of 0, 3, 6, and 9% and the CNFs at 0, 0.3, 0.4, and 0.5%. The dependent variables considered were penetration at 25 °C, softening point, viscosity at 135 °C, PI, PVN, elastic recovery, adhesion force, G* at 60 °C and 0.1 Hz, G* at 60 °C and 10 Hz, and G*/sinδ at 60 °C and 10 Hz. The selection of the variables and their experimental ranges was based on the preliminary studies. At least three specimens’ replicates were prepared for each binder per test.

To evaluate the individual and interaction effects of the independent variables on the dependent variables (responses) and to check the appropriateness of the selected models, analysis of variance (ANOVA) was conducted. ANOVA measured and confirmed the suitability of the proposed models and the significance of each variable. The correlation of coefficient (R^2^) was used to assess the fitness of the experimental data obtained from the laboratory and selected models. In order to ensure the probability within the typical confidence level of 95%, Fisher’s F test was conducted.

## 3. Results and Discussions

### 3.1. Conventional Physical Properties

#### 3.1.1. Penetration Test Results

**Figure 2:**
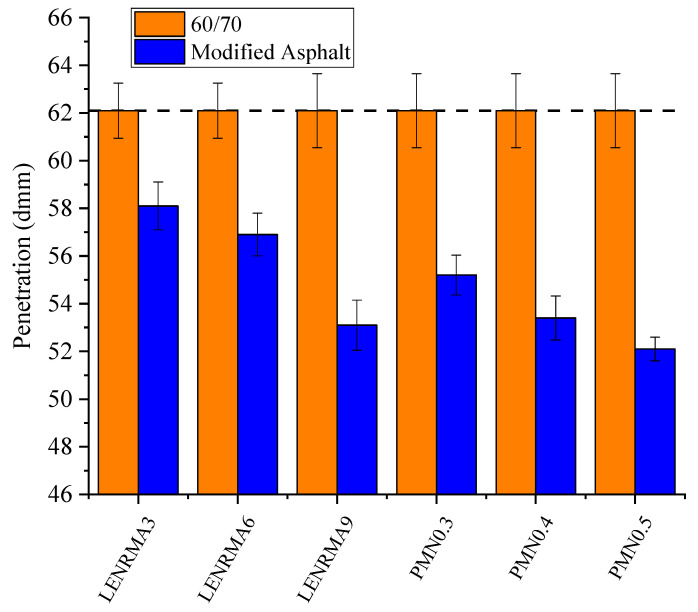
Penetration of all binders.

The hardness and consistency of asphalt at intermediate temperatures can be identified by the penetration test. Figure 2 shows the penetration values of base and modified asphalt binders at various LENR and CNFs concentrations. Overall, it can be seen that the addition of LENR and CNFs shows further improvement to the penetration characteristics compared to base asphalt which indicates the significant improvement in the deformation resistance of modified binders. The penetration keeps decreasing as the content of LENR and CNF increases. This indicates the incorporation of LENR into the base asphalt results in stiffer binders, which could be due to the stiff nature of rubber. Moreover, the addition of CNFs into LENR-modified asphalt resulted in improvement of the hardness and stiffness of binders. This finding could be due to the uniform physical dispersion of CNFs in the LENR-modified asphalt matrix that results in a hard binder. It can also be noted that the lowest penetration value of LENR-modified binders found at 9% was 14.4% lower than the penetration value of base asphalt. On the other hand, PMN0.5 exhibited the lowest penetration value among all tested binders in this study with 16% improvement compared to base bitumen. These findings indicate that LENR and/or CNFs are suitable modifiers to improve the consistency of base binders to be applied at the intermediate temperatures.

#### 3.1.2. Softening Point Test Results

**Figure 3:**
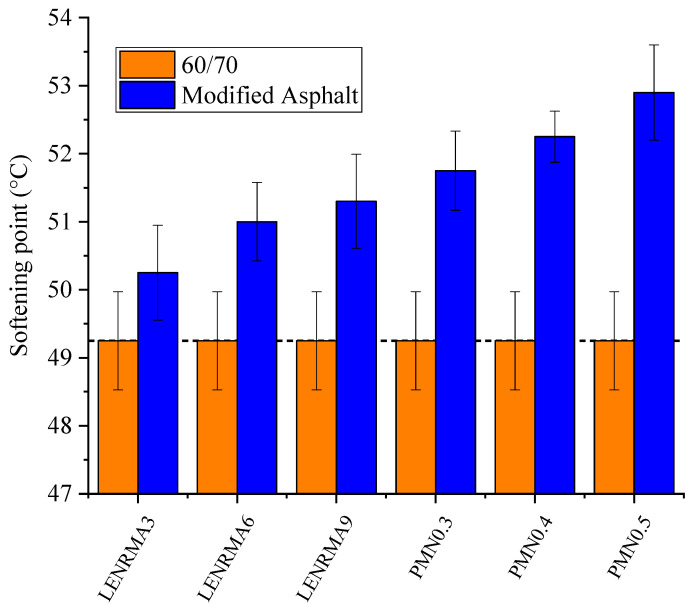
Softening point of all binders.

Figure 3 shows the softening point test results of base and modified binders. It can be noticed that the softening point increases as the content of LENR and CNFs increases. All modified binders exhibited softening point values higher than that for base asphalt. This could be ascribed to the stiffness of rubber and the effects of CNF additives that improved the temperature sensitivity resistance of binders. LENRMA9.0 showed the highest softening value among LENR modified binders with an increase of 2 °C compared to base asphalt, while PMN0.5 showed the highest softening point among all tested binders with 3.65 °C improvement. These findings could be due to the high-temperature resistance of rubber and CNFs that delayed the softening of base asphalt. These results are consistent with the findings from the penetration test which also indicates the improvement of binder consistency due to the addition of LENR and CNFs to be used for high-temperature pavement applications.

#### 3.1.3. Rotational Viscosity Test Results

**Figure 4:**
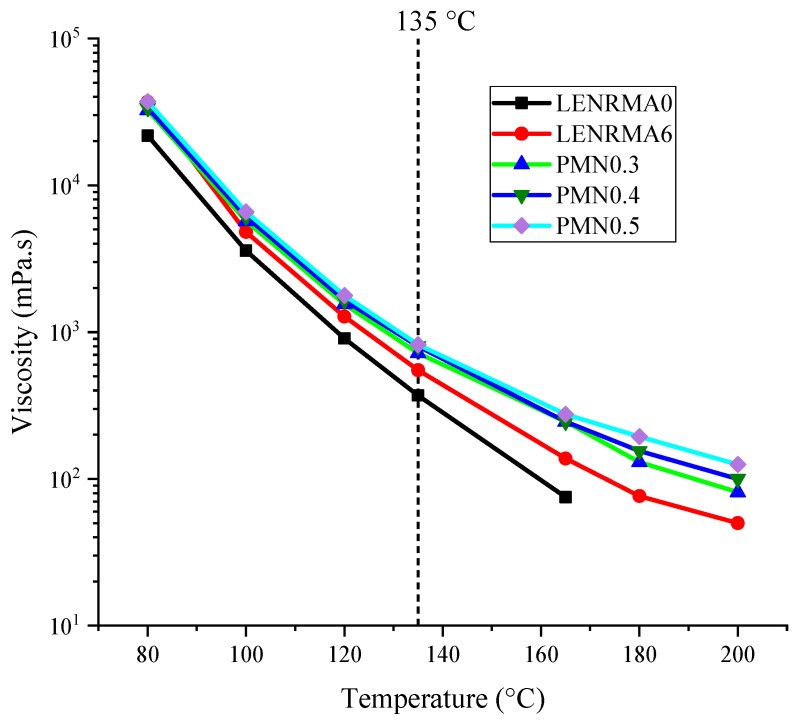
Rotational viscosity of all binders.

**Figure 5:**
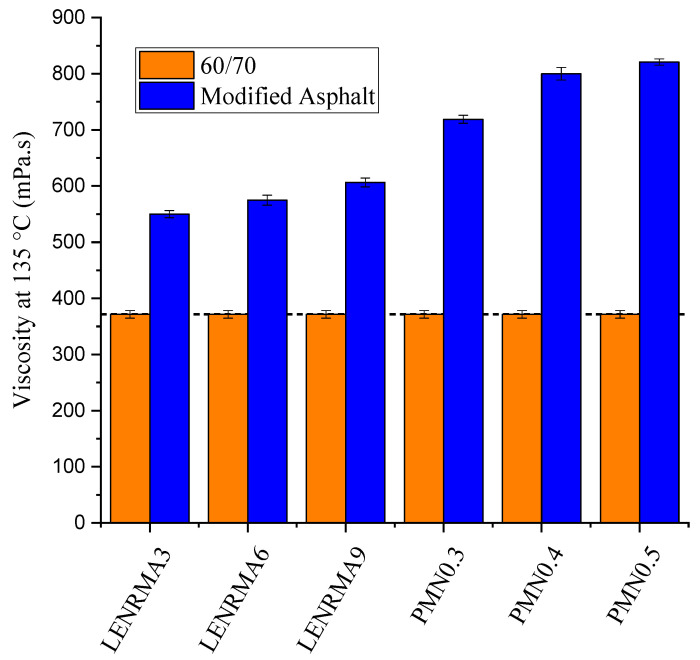
Rotational viscosity of all binders at 135 °C.

The rotational viscosity results of the base and modified binders over the temperature range of 80 to 200 °C are shown in Figure 4. Overall, it can be stated that the viscosity of base and modified binders always decreases as the test temperature increases. This could be due to the reduction of the binder cohesion, particularly at a high temperature which leads to the softened binder. It can also be noted that all the modified binders showed viscosity higher than base asphalt and the viscosity of the modified asphalt increased as the content of LENR and CNFs increased. Such improvement could be attributed to the shear resistance of rubber and CNF additives that reinforce the shear strength of base asphalt and came up with high viscous binders. A variation of viscosity among various binders that tested in this study became more obvious at low temperatures which could be due to the Newtonian properties of binders. Similar improvement in the viscosity of binders that were modified with ENR and nanoalumina (Al_2_O_3_) were observed in the literature [21]. PMN0.5 showed the highest viscosity among all tested binders over the test temperature range, which could be justified due to the shear resistance of nanoparticles that led to stiffer modified binders. This finding is in agreement with the physical properties of the CNF-modified binders aforementioned. In order to compare the viscosity of the modified binders to the base asphalt at specific temperatures, Figure 5 exhibits the viscosity of all tested binders at 135 °C. It can be seen that all modified binders have a viscosity value higher than base asphalt due to the addition of LENR and CNFs additives. Lowest viscosity of modified binders was found at LENRMA3 with an improvement of 48% compared to base asphalt at 135 °C. On the other hand, the highest improved viscosity among modified binders was observed for the PMN0.5 binder with an improvement of 449.5 mPas compared to base asphalt. It can be said that the viscosity and flow resistance of base asphalt binders were enhanced somewhat as the LENR and CNFs were added, indicating their suitability for high-temperature applications.

### 3.2. Temperature Susceptibility

**Figure 6:**
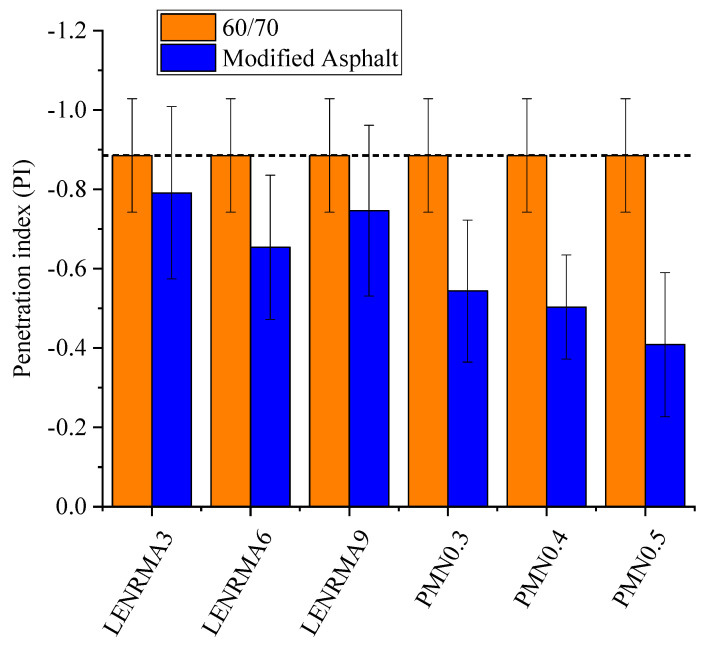
Penetration index of all binders.

**Figure 7:**
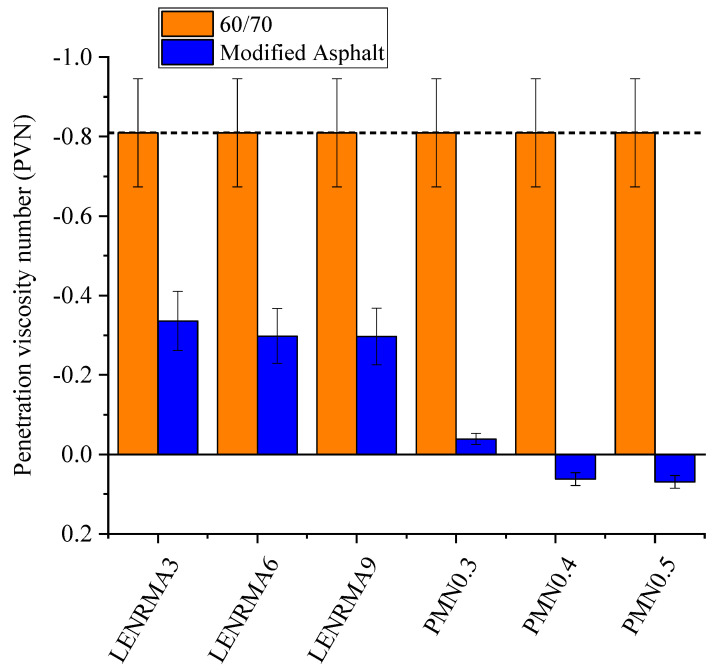
Penetration viscosity number of all binders.

Temperature susceptibility of binders can be determined by the penetration index (PI) value adopted from the penetration and softening point test results. Penetration viscosity number (PVN) is also another indicator of temperature susceptibility which can be obtained from the viscosity at 135 °C and penetration value at 25 °C. Figure 6 and Figure 7 show the PI and PVN results of the base and modified asphalt binders, respectively. It can be observed that the addition of LENR content increases the PI value which indicates a decrease in the temperature susceptibility. Such effects could be ascribed to the elastic properties of rubber that enhance the temperature resistance of the base binder. However, LENRMA9 shows more susceptibility to temperature than LENRMA6, which indicates low compatibility between the asphalt and LENR as a polymer. On the other hand, the addition of CNFs to LENRMA increases the PI value of the binder. This could be attributed to the improvement of the chemical structure of LENR-modified binders and the binders becoming stiffer to resist the temperature. Thus, as the content of CNFs increases, the temperature susceptibility of the modified binders was decreased and the lowest temperature susceptibility among all tested binders was shown for the PMN0.5 binder. Similar behaviour was found from the PVN curve which supports the findings of PI values that CNFs significantly improved the compatibility and temperature resistance of LENR-modified binders. It can be said that all modified binders tested in this study are suitable to be used for asphalt pavement construction.

### 3.3. Elastic Recovery

**Figure 8:**
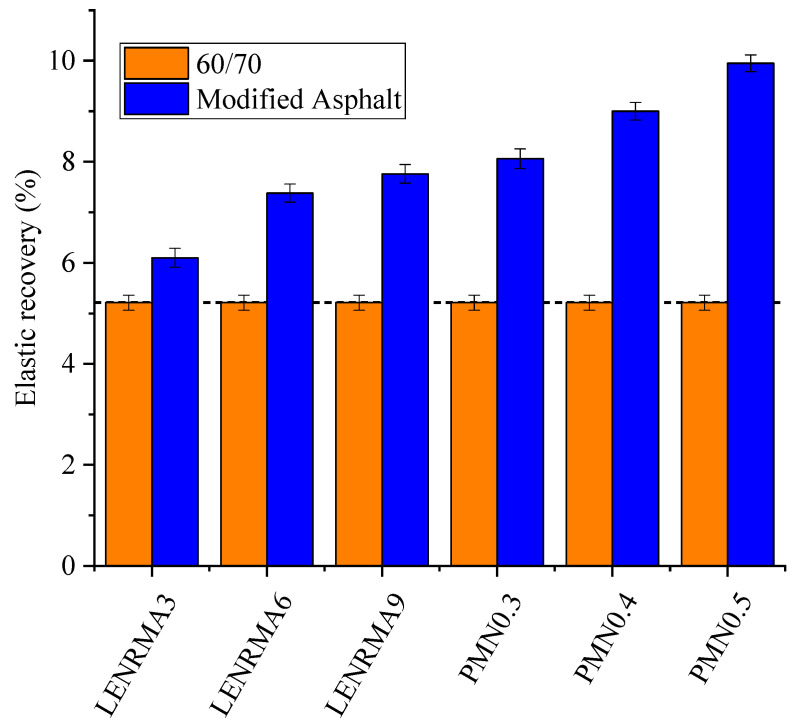
Elastic recovery test results of modified binders.

The determination of the elastic recovery value of the binder is crucial to indicate the permanent deformation behaviour. The effect of LENR and CNFs on the elastic recovery of the base and modified binders is shown in Figure 8. Overall, it can be observed that all modified binders with LENR and CNFs showed higher elastic recovery compared to base asphalt. These improvements in the elasticity could be ascribed to the elastic nature of rubber and the physical interaction between the rubber and nanoparticles that results in such higher elasticity. The recovery percentage of the LENRMA is almost linear with the increase of LENR content and LENRMA9 exhibits the highest elastic recovery among all tested rubberized binders. This indicates that higher LENR content improves the elastic recoveries of binder which is due to the higher elastic properties of natural rubber. Based on Nejad et al. [15], the same trend was found whereby increasing the content of crumb rubber in modified asphalt, the higher the viscosity recovery percentage was observed. On the other hand, the recovery significantly increased with the addition of 0.4 and 0.5% of CNFs, indicating a great improvement in the elasticity of the binders. This could be ascribed to the elastic networks constructed due to the interaction of CNFs in the asphalt matrix. The lowest elastic recovery among modified binders showed for LENRMA3 with improvement of 0.9% compared to the base asphalt, however the highest elastic recovery was found at 0.5% CNFs with an improvement of 91% compared to the unmodified binder. Based on the findings from elastic recovery results, it can be indicated that all the modified binders that were tested in this study could perform well against the elastic deformation at intermediate temperatures.

### 3.4. Atomic Force Microscopy

**Figure 9:**
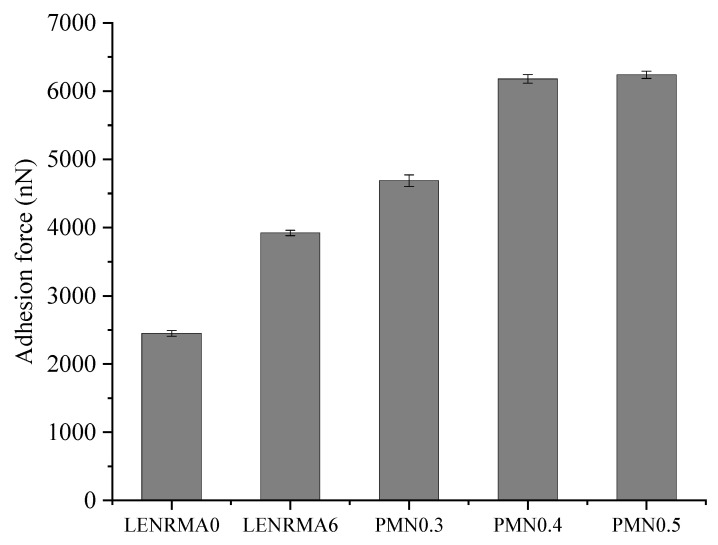
Adhesion by AFM force for all binders.

Figure 9 shows the adhesion forces of CNF-modified binders mixed with 6% of LENR and compared to base asphalt and LENRMA6 binders. It can be seen that all binders modified with CNFs showed higher adhesion forces compared to the base and rubberized binders. Such improvement in adhesion properties could be due to the higher stiffness and elastic properties of composite CNFs and natural rubber that resulted in adequate tensile strength and desired adhesion properties. The adhesion forces increased as the content of CNFs increased with the highest force occurring at 5% CNF content. PMN0.5 showed 154.7% and 59.14% higher adhesion forces compared to the base asphalt and LENRMA6 binders, respectively. This finding also proves the improvement of the bonding strength of modified binders which could be due to the higher modulus properties of binders that are modified with CNFs. However, the binders with CNF content of 0.4% and 0.5% have no significant differences. The significant difference of the modified binder to the control shows the potential indication of a good adhesion between aggregate and the modified binder that could occur. This could be an indicator for adequate moisture damage resistance in the level of asphalt mixtures. Therefore, it can be said that in terms of moisture-induced damage, the modified binders that were developed in this experiment could exhibit a good potential for future field application.

### 3.5. Rheological Properties

#### 3.5.1. Isochronal Plots

**Figure 10:**
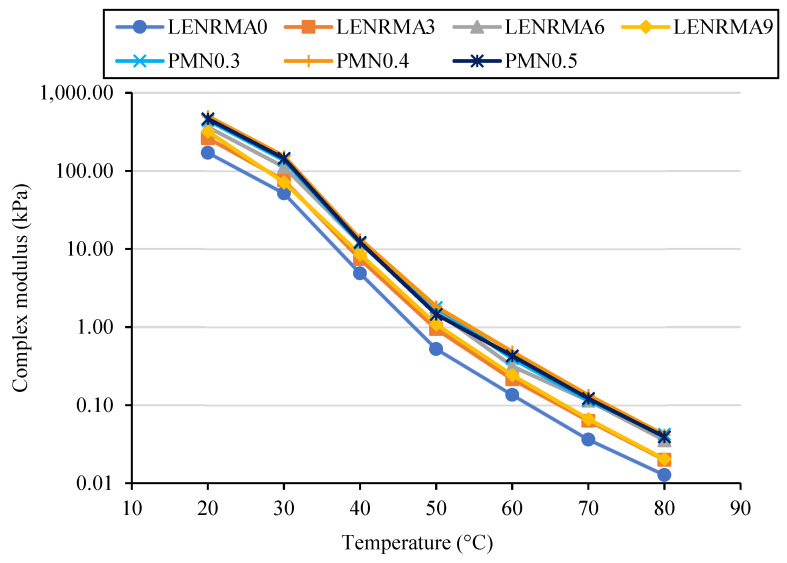
Complex modulus versus temperature at 0.1 Hz of binders (isochronal plots).

**Figure 11:**
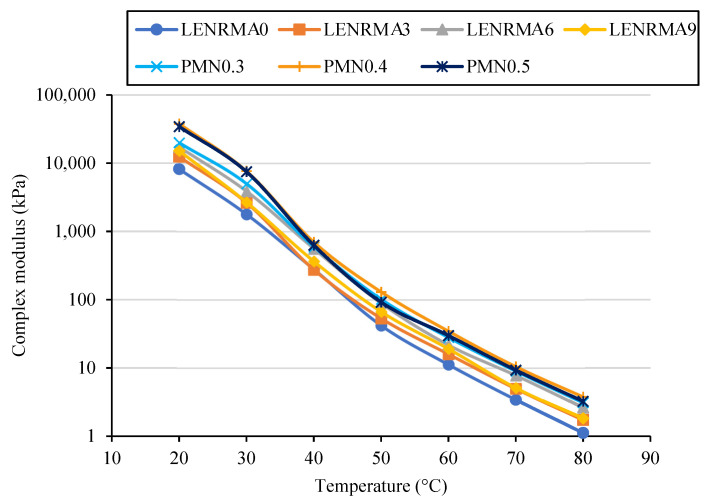
Complex modulus versus temperature at 10 Hz of binders (isochronal plots).

The effect of temperature on the complex modulus (G*) of base, LENRMAs, and PMN-modified binders at a temperature range of 20 to 80 °C under 0.1 Hz and 10 Hz frequencies are presented in Figure 10 and Figure 11, respectively. Regardless of the frequency and concentrations of rubber and nanoparticles, G* of the base and modified binders decreased with the increase of the testing temperature. Such observation is well-known due to the reduction of asphalt cohesion at higher temperatures which results in softer binders. It can be observed that G* of LENRMA binders increases as the rubber content increases up to 6% and then starts to decrease. This behaviour was shown along the test temperature range at both frequencies of 0.1 and 10 Hz. Such findings could be due to the reduction of compatibility between the rubber and asphalt beyond 6% which results in softer binders. That means the sensitivity of binders modified with more than 6% LENR become more temperature susceptible. This finding is consistent with results reported in the literature about using tire pyrolysis rubber in asphalt binders [14]. In this study, LENRMA6 showed a more pronounced increase in G* and improved temperature susceptibility. Therefore, LENRMA6 was used to study the effect of CNFs on the rheological performance of LENRMA binders.

The reduction of temperature susceptibility can be identified by the decrease in the slope of complex modulus isochrones. Every binder has different rheological behaviour which is shown by the changing of the G*. From Figure 10 and Figure 11, it can be noticed that PMNs show identical trends. However, PMN0.4 shows a more pronounced increase and improvement of temperature susceptibility among all binders at both frequencies that were used in this research. These results could be attributed to the aggregation of CNFs at high concentration and the nonuniform elastic matrix in the asphalt binder that leads to a lower complex modulus. Generally, the isochronal plots exhibited a trend that is similar to other studies done before [20]. It can be concluded that due to the addition of LENR and CNFs, the complex modulus significantly increases and improves the viscoelastic properties which leads to improving the stiffness of the modified binders. The increase of G* of the binder is the result of the different flow and deformation properties of the binders. The increase of binder stiffness at high temperature and reduction at low temperature due to the addition of CNFs provides an improvement for temperature susceptibility and shows the capacity to resist against both rutting and cracking.

#### 3.5.2. Master Curves

**Figure 12:**
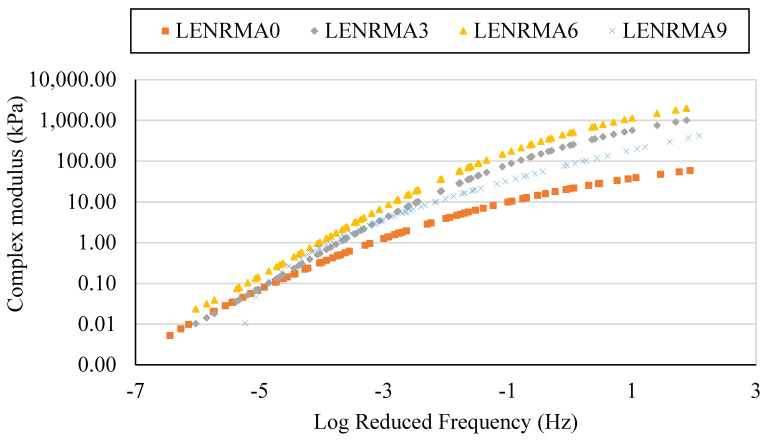
Complex modulus master curve for LENRMAs at a ref. temperature of 25 °C.

**Figure 13:**
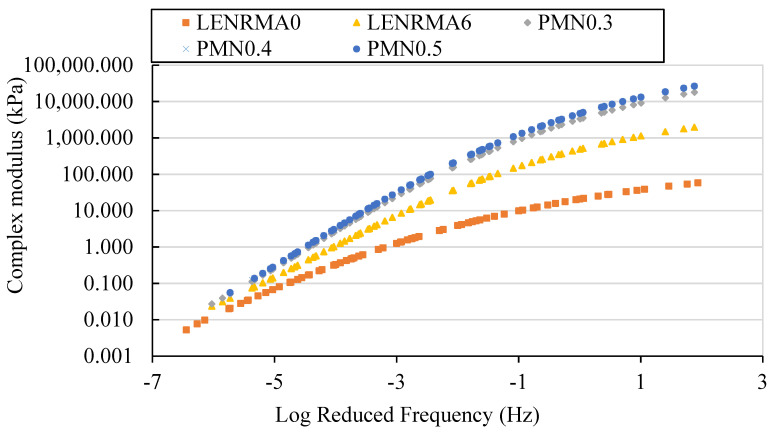
Complex modulus master curve for PMNs at a ref. temperature of 25 °C.

Overall, master curves can represent the material behaviour and properties depending on the time and frequency scale that is more similar to the results obtained by conducting a normal laboratory test. Developing a master curve mainly depends on the equilibrium between temperature and time, which is known as the time–temperature superposition principle. The master curves are obtained in this study according to the Williams–Landel–Ferry (WLF) equation. The complex modulus master curves for LENRMAs and PMNs at a reference temperature of 25 °C are presented in Figure 12 and Figure 13, respectively. From Figure 12, it can be seen that all developed master curves for base and rubberized asphalt binders show a similar trend at the range of frequencies that have been considered in this study. It can be also noticed that, at low frequency (high testing temperatures), complex modulus values of rubberized binders increased compared to base asphalt which can be attributed to the reduction of temperature susceptibility, increasing of viscosity and stiffness of the LENRMA binders as found from the temperature susceptibility and consistency tests. Compared with the effects of ENR as a modifier, it can be stated that LENR shows a similar trend of ENR at different concentrations from 3% to 9% [20]. The overall trend of complex modulus master curves increased subsequently as the modifier content and log frequency increased. This finding indicates the improvement of permanent deformation resistance of LENR-modified binders compared to base asphalt binder resistance, which could be due to the elastic network that has been formed in the asphalt matrix with the addition of LENR. A similar behaviour was found in a previous study of SBS-modified asphalt [41].

The effects of CNFs on the master curves of the optimum rubberized binder are presented in Figure 13 against the master curves of the base and LENRMA binders as control. It can be observed that there are significant changes of complex modulus master curves towards higher value as the CNF content increases over the wide range of the frequencies that have been considered in this study compared to the base asphalt as the reference. This indicates an improvement in the stiffness of nano-modified rubberized binders which leads to higher shear strength and permanent deformation resistance at various temperatures and frequency of traffic loading. Such improvement could be ascribed to the physical and chemical interaction of CNFs with LENR in the asphalt matrix that results in the improvement of the binder elasticity and stiffness to stand for higher temperatures and frequency loading. The most significant shifting of master curves of the complex modulus occurs at low log reduced frequency. PMN0.4 and PMN0.5 show nearly identical results with small shifting at log reduced frequency of more than −2. This finding could be due to the aggregation of CNFs at the high content level which is consistent with the results obtained from the literature for the application of nanomaterials as a modifier for asphalt binders [3,27]. Similar behaviour of CNFs at the high content level was also noticed in this study for viscosity and adhesion tests results.

#### 3.5.3. SHRP Rutting Parameter

**Figure 14:**
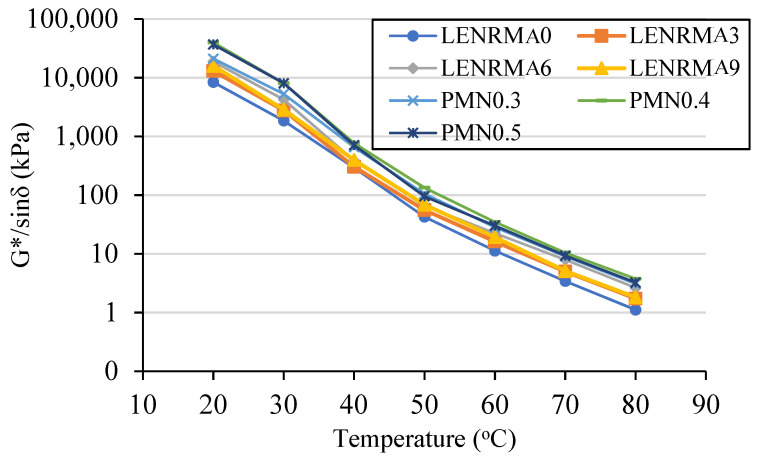
Effect of temperature on the rutting parameters (G*/sinδ).

According to the Strategic Highway Research Program (SHRP) specifications, the minimum rutting parameter (G*/sinδ) for asphalt binder at unaged condition is 1 kPa to perform well against permanent deformation at high temperatures. G*/sinδ has been identified as an adequate indicator to predict the rutting resistance of base and modified asphalt binders. Figure 14 presents the relationship of the rutting parameter versus the testing temperatures for LENRMA and CNF-modified asphalt binders against base asphalt at a frequency of 10 Hz. Overall, it can be noted that G*/sinδ of the base and modified binders decreased with the increase of the testing temperature. This is due to the reduction in the cohesion of binders which become more viscous and less elastic at high temperatures. It can also be observed that all modified binders showed a significant improvement in the G*/sinδ along the testing temperatures range compared to base asphalt. It can also be seen that the G*/sinδ increases as the LENR content increases up to 6%, which could be due to the compatibility of LENR with asphalt binder that results in more elastic binders to resist the deformation. LENRMA6 has the highest rutting parameter among all LENRMAs. On the other hand, CNFs showed a significant enhancement in the rutting parameter of rubberized binders and the G*/sinδ increased as the CNFs increased. Such results could lead to adequate stiffness and rutting deformation resistance at a temperature range of 20 to 80 °C. It can also be stated that the composite of LENRMA and CNFs has shown a significant improvement in the rutting distress resistance and the modified binder that exhibited the best performance against rutting among all tested binders was PMN0.4 with the highest G*/sinδ value. This could be due to the interaction effects of rubber and CNFs that constructs a well-connected elastic network in the asphalt matrix which results in higher permanent deformation resistance compared to those individually modified with LENR or CNFs. The same trends were observed in the literature for the effects of ENR content up to 6% on rutting resistance of asphalt which resulted in increasing the stiffness and improvement of rutting resistance [20,21,34].

### 3.6. Statistical Analysis Results

Table 3 exhibits the summary of the statistical analysis of physical, thermal, and rheological properties of asphalt binder modified with LENR at 0, 3, 6, and 9% LENR. It can be noticed that the *p*-value of most of the dependent variables are more than 0.05 which indicates the effects of LENR on the physical and rheological properties of asphalt binder are not statistically significant. In contrast, it can be observed that the *p*-value of most of the dependent variables, as shown in Table 4, are less than 0.05 which indicates the effect of CNFs at 0, 0.3, 0.4, and 0.5% on the physical, adhesion, and rheological properties of base and LENR-modified asphalt binders is statistically significant. The degree of correlation between the independent and dependent variables can be indicated by correlation coefficient (R^2^). From Table 3, it can be seen that the highest R^2^ value showed for penetration value of 0.9644 which indicates that almost 96.44% of the change in the penetration value of base asphalt was due to the addition LENR, which reflects the highest effects of LENR on the penetration properties of base asphalt. On the other hand, the lowest R^2^ value of 0.6913 showed for complex modulus at 0.1 Hz, which indicates that LENR only contributed to 69% of the change in the stiffness of base asphalt. From Table 4, it can be noted that all R^2^ values are more than 0.84 and overall R^2^ values are higher than that for the same responses in Table 3. This reflects the strong effect of CNFs on the physical, adhesion, and rheological properties of base and rubberized asphalt compared to only using LENR. It can be said that CNFs contribute to more than 95% of physical and adhesion properties improvement and about 84% of the rheological performance enhancement. Furthermore, the lower values of standard deviation and correlation of variance compared to the mean values clearly explain that the experimental results were fitted with the suggested models used with less variability.

## 4. Conclusions and Future Directions

The objective of this research was to investigate the physical and rheological properties of base asphalt, LENR-modified asphalt, and LENR/CNFs composite-modified asphalt. The conventional tests (penetration, softening point, viscosity, and elastic recovery tests), microstructure, and DSR tests have been conducted. The base asphalt was mixed with 3, 6, and 9% of LENR by weight of asphalt. Furthermore, the effects of CNFs at concentrations of 0.3, 0.4, and 0.5% by weight of asphalt on the performance of LENRMA binders containing 6% LENR has been investigated. Based on the test results and analysis, the following conclusions can be drawn:

1. Results of physical properties showed that the addition of LENR and composite LENR/CNFs into base asphalt led to a decrease in penetration and temperature susceptibility and increase in softening points, which indicates the improvement of base and rubberized asphalt consistency. PMN0.5 showed the best consistency among all tested binders.
2. The highest elasticity recovery was also achieved for the PMN0.5 binder among all tested binders, which could reflect the possible enhancement of the tensile properties of modified binders. Binders modified with 0.4 and 0.5% of CNF showed significant high adhesion performance among all tested binders.
3. Addition of 0.3 to 0.5% of CNFs into LENR-modified asphalt exhibited a significant increase in the adhesion forces which reflects the improvement of bonding and interlocking strength that is expected between modified binders and aggregate.
4. The addition of 0.3 to 0.5% of CNFs into rubberized asphalt enhanced the viscosity and complex modulus of LENR-modified binders with an increase in CNFs, which indicates the improvement of the stiffness and hardness of the modified binders.
5. Binders modified with 6% LENR and 0.4% CNF content provided the best performance of stiffness and temperature susceptibility represented by the complex modulus value over the wide range of testing temperatures.
6. SHRP rutting parameter showed an improvement due to the addition of CNFs into LENR-modified binders. PMN0.4 exhibited the highest G*/sinδ value of 3.25 kPa at 80 °C compared to 1.1 kPa for LENRMB0, indicating the higher rutting resistance of LENR/CNFs composite binders at high-temperature applications.
7. Statistical analysis presented that the effects of CNFs on the physical, adhesion, and rheological properties of base and LENR-modified asphalt is statistically significant within 95% confidence of interval.
8. Correlation coefficient (R^2^) obtained from the statistical analysis for the effects of CNFs showed values between 0.84 and 0.99, which reflects that at least 84% of the improvement in the performance of asphalt was due to the addition of CNFs.
9. In conclusion, the contents of LENR and CNFs that showed the appropriate physical, adhesion, and rheological performance in this study and can be recommended as an optimum are 6% of LENR and 0.4% CNFs by weight of base asphalt.
10. It is recommended that further indepth investigations on the storage stability, physicochemical, morphological, and thermal properties of CNFs/LENR composite asphalt binders to be conducted.
11. The low-temperature performance evaluation of CNFs/LENR composite asphalt binders can be performed in the regions where the low-temperature failure is a major concern.
12. Life cycle cost analysis is recommended to be studied to evaluate the long-term performance of the LENR and LENR/CNFs composite asphalt binders compared to the base asphalt binder.

## Figures and Tables

**Table 1:** Properties of the based asphalt, LENR polymer, and CNFs.

Material	Properties	Standard Method	Value
LENR	Specific gravity	-	0.50
Asphalt	Specific Gravity	ASTM D70	1.03
	Penetration @ 25 °C	ASTM D5	62.1
	Softening point (°C)	ASTM D36	49.25
	Viscosity (Pa.s) @ 135 °C	ASTM D4402	0.37
	Ductility (cm) @ 25 °C	ASTM D113	≥100
CNFs	Diameter (nm)	-	200
	Length (μm)	-	50–200
	Apparent density (kg/m^3^)	-	30–300

**Table 2:** Properties of the base asphalt, LENR polymer, and CNFs.

Binders	IDs
Base asphalt	LENRMA0
Base asphalt + 3% LENR	LENRMA3
Base asphalt + 6% LENR	LENRMA6
Base asphalt + 9% LENR	LENRMA9
Base asphalt + 6% LENR + 0.3% CNF	PMN0.3
Base asphalt + 6% LENR + 0.4% CNF	PMN0.4
Base asphalt + 6% LENR + 0.5% CNF	PMN0.5

**Table 3:** Analysis of variance results summary at various LENR contents.

Parameter	Sum of Squares	df	Mean Square	F-Value Square	*p*-Value	R^2^	Std. Dev.	C.V. %
Penetration value @ 25 °C	39.76	1	39.76	54.17	0.018	0.9644	0.86	1.49
Softening point	2.38	1	2.38	38.24	0.0252	0.9503	0.25	0.49
Viscosity @ 135 °C	0.11	1	0.11	6.54	0.1249	0.7658	0.13	2.12
Penetration index	0.024	2	0.012	3.24	0.3655	0.8664	0.061	7.91
Penetration viscosity number	0.18	2	0.090	11.32	0.2056	0.9577	0.089	20.50
Elastic recovery	3.99	1	3.99	53.80	0.0181	0.9642	0.27	4.12
G*@ 60 °C and 0.1 Hz	31,804.39	2	15,902.19	1.12	0.5556	0.6913	119.16	46.69
G*@ 60 °C and 10 Hz	1.301 × 10^8^	2	6.503 × 10^7^	1.16	0.5493	0.6983	7496.2	39.40
G*/sinδ @60 °C and 10 Hz	1.353 × 10^8^	2	6.766 × 10^7^	1.17	0.5476	0.700	7613.4	39.47

**Table 4:** Analysis of variance results summary at various CNFs contents.

Parameter.	Sum of Squares	df	Mean Square	F-Value Square	*p*-Value	R^2^	Std. Dev.	C.V. %
Penetration value @ 25 °C	58.84	1	58.84	188.27	0.0053	0.9895	0.56	1.00
Softening point	7.58	1	7.58	236.74	0.0042	0.9916	0.18	0.35
Viscosity @ 135 °C	1.259 × 10^5^	1	1.259 × 10^5^	49.55	0.0196	0.9612	50.40	7.44
Penetration index	0.13	1	0.13	112.01	0.0088	0.9825	0.034	5.76
Penetration viscosity number	0.54	2	0.27	10,552.83	0.0069	0.9999	0.005	2.81
Elastic recovery	12.58	1	12.58	7.044 × 10^5^	<0.0001	0.9999	0.003	0.052
Adhesion force	9.137 × 10^6^	1	9.137 × 10^6^	53.10	0.0183	0.9637	414.81	8.48
G*@ 60 °C and 0.1 Hz	62,384.55	1	62,384.55	13.21	0.0681	0.8685	68.72	19.02
G*@ 60 °C and 10 Hz	2.608 × 10^8^	1	2.608 × 10^8^	10.79	0.0815	0.8437	4915	18.99
G*/sinδ @60 °C and 10 Hz	2.705 × 10^8^	1	2.705 × 10^8^	10.94	0.0805	0.8454	4973	18.93

## Data Availability

The data presented in this study are available on request from the corresponding author.
